# Sinonasal mucosal melanoma with smooth muscle differentiation: a potential pathological diagnostic pitfall

**DOI:** 10.1186/s13000-022-01280-x

**Published:** 2022-12-23

**Authors:** Hao Tang, Yutao He, Ying Chen, Wenfeng Xu, Yujuan Xu, Xianyun Li, Deyu Guo

**Affiliations:** 1 Department of Pathology, Guiqian International General Hospital, Guiyang, Guizhou Province China; 2 School of Public Health, Medical College, Wuhan University of Science and Technology, Wuhan, Hubei Province China

**Keywords:** Sinonasal, Melanoma, Smooth muscle differentiation, Pitfall

## Abstract

**Background:**

Sinonasal mucosal melanoma (SNMM) is a rare malignant melanoma originating from melanocytes derived from multipotent neural crest cells. Its incidence accounts for less than 1 % of all malignant melanomas, with five-year survival rate about 25 %. Occasionally, it is incredibly formidable to make a compelling diagnosis when malignant melanoma with other diverse differentiation.

**Case presentation:**

Herein, we presented a 54-year-old male case of SNMM with smooth muscle differentiation, defined by histopathology and positive immunostaining for the smooth muscle specific markers of a-SMA, H-caldesmon, calponin and Desmin, as well as specific melanocyte markers of HMB-45, Melan-A, SOX10, and PNL2.

**Conclusions:**

Mucosal melanoma with smooth muscle differentiation is remarkably infrequent, and reported only 4 cases to date. It would be a potential pathological diagnostic pitfall. It is important to understand this variation of malignant melanoma for avoiding misdiagnosis.

## Introduction

Sinonasal mucosal melanoma (SNMM) is a rare and fatal aggressive malignancy with an estimated incidence of less than 1 % of all malignant melanomas (MMs), with the proportion of about 4 % of all sinonasal tumors, and its overall 5-year survival is only approximately 25 % [1, 2]. It derives from melanocytes on the mucosal surface of the nasal cavity, whereas melanocytes originate from multipotent neural crest cells and pass through mesenchyme of embryo to the skin, eye, mucosal membranes and leptomeninges [3, 4]. It was identified that up to 83 % of patients with SNMM were accompanied by intraepithelial melanocytic lesion (including melanocytic hyperplasia and melanoma in situ), related to mucosal invasive melanoma [5]. Unlike cutaneous and uveal melanomas, formaldehyde but rather exposure in ultraviolet light is regarded as a risk factor for SNMM [6]. It always was misconstrued or missed because of having few early specific symptoms [7]. Occasionally, it presents nasal obstruction and in particular epistaxis at early stage [7].

Divergent differentiation of MM is a scarce situation which characterizes by the manifestations of histomorphologically, immunohistochemically, and/or ultrastructurally legible nonmelanocytic cellula or cellular compositions in melanomas [8]. Multiple kinds of heterologous elements could be observed in MM, such as schwannian and perineurial, fibroblastic/myofibroblastic, neuroendocrine, rhabdomyosarcomatous, osteocartilaginous, smooth muscle, ganglionic, ganglioneuroblastic, and epithelial elements [9]. Their histomorphological multiformity invariably results in the diagnosis complexity, with numerous differential diagnoses [10].

Herein, we presented an exceptional case of SNMM with histomorphological and immunochemical proofs of smooth muscle differentiation, which would be a potential conundrum or pitfall of pathological diagnosis.

## Case presentation

**Fig. 1:**
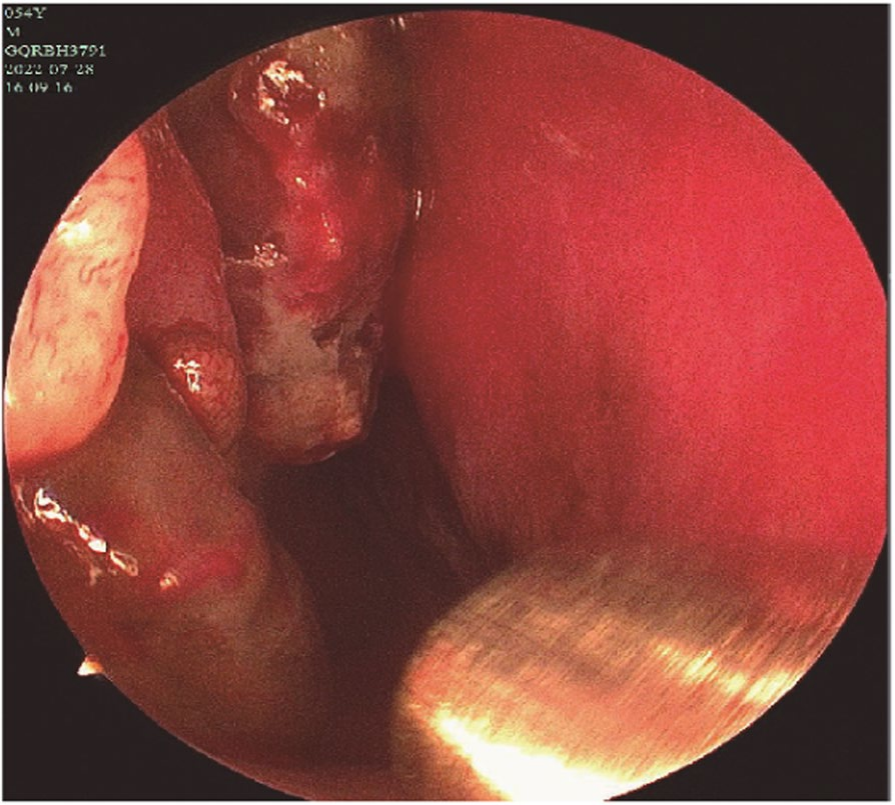
Flexible nasolaryngoscope image of old bleeding in the right nasal cavity and a pigmented mass in the right inferior meatus and middle turbinate

**Fig. 2:**
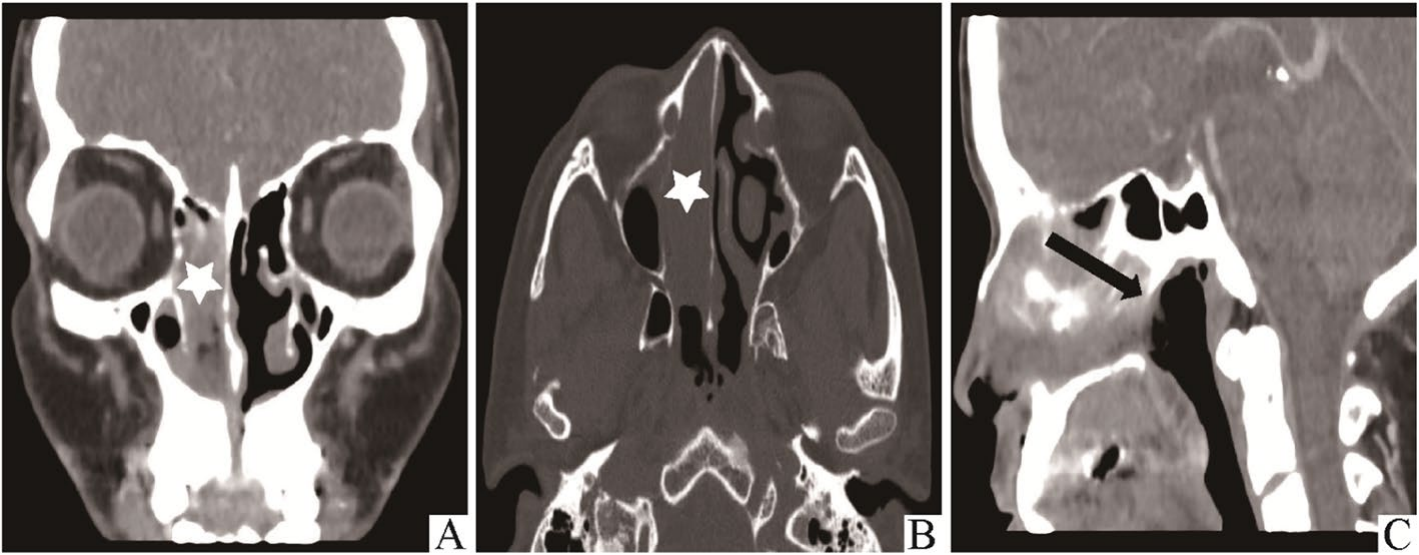
Coronal section of high-resolution computed tomography (CT) showed an inhomogeneously enhancing distended mass in the right nasal cavity (white asterisk) (**A**). Axial view exhibited the lesion extension to the right maxillary sinus (white asterisk) and a contralateral nasal septum deviation after breach (**B**). Sagittal view demonstrated lesion extending posteriorly beyond the inner nostril (black arrow) (**C**)

A 54-year-old male patient complained of symptoms of right nasal obstruction for 3 months and intermittent epistaxis for 1 month. A flexible nasolaryngoscopy was performed which demonstrated old bleeding in the right nasal cavity, and a pigmented mass in the right inferior meatus as well as the middle turbinate (Fig. 1). The mass could bleed easily when touched. High-resolution computed tomography (CT) scan of the paranasal sinuses disclosed a solid, large, irregular mass measuring 57.5 mm × 48.5 mm × 22.3 mm in the right nasal cavity with surrounding bone destruction, suggesting malignancy (Fig. 2). In the meantime, for the purpose of in-depth diagnosis and treatment of this patient, positron emission tomography/computed tomography (PET/CT) examination was executed and no any other positive sign in the body was suggested. Subsequently, an endoscopic resection was undergone.

### Histopathology

Grossly, the specimen consisted of multiple pieces of fleshy, gray-white to brown soft tissue fragments with a total size of 65.0 mm × 40.0 mm × 10.0 mm. Its cut surfaces were off-white to brown with a medium texture.

**Fig. 3:**
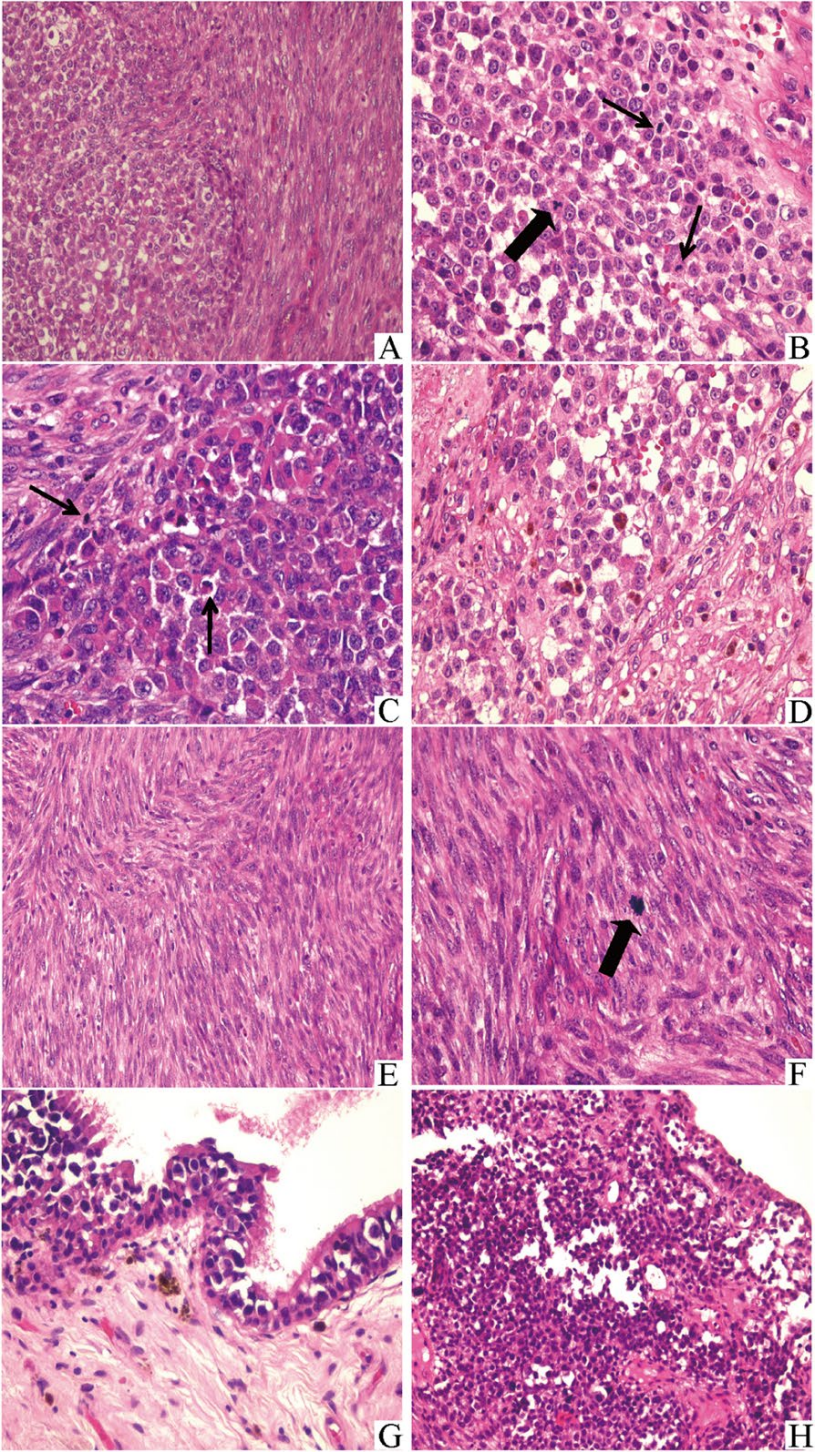
Histopathologically, tumor cells consisted of a biphasic component morphology of epithelioid or rhabdoid and spindle-shaped (**A**, × 200). In the epithelioid or rhabdoid areas, there were numerous mitotic figures (20 mitoses per 10 high-power fields) (thin arrow), even atypical pathological mitoses (thick arrow) (**B** and **C**, × 400). A few of these neoplastic cells contained intracytoplasmic pigments (**D**, × 400). In the spindle areas, tumor cells had a fusiform morphology with a fascicular growth pattern (**E**, × 200), and showed fusiform nuclei, with dispersed chromatin, infrequent eosinophilic cytoplasm, numerous mitotic figures (17 mitoses per 10 high-power fields) and 1 or 2 prominent nucleoli (**F**, × 400). Melanoma in situ in the superficial of nasal mucosa and its transition to the epithelioid area of the tumor in submucosa were observed (**H**, × 200)

Microscopically, a biphasic component morphology consisting of epithelioid and spindle-shaped parts was observed at low magnification (Fig. 3A). In the epithelioid areas, the tumor cells grew in nodular form and appeared epithelioid or rhabdoid features. Tumor cells were polygonal or oval in shape, medium to large in size, and distributed in sheets, with prominent nucleoli, prominent mitoses (thin arrow) (Fig. 3B and C), and pathological mitoses (thick arrow) (Fig. 3B). The intracellular pigment was observed in a small percentage of neoplastic cells (Fig. 3D). While in the spindle-shaped areas, the tumor cells were arranged in fascicles (Fig. 3E). These spindle-shaped tumor cells were characterized by infrequent eosinophilic cytoplasm, fusiform nuclei, dispersed chromatin, 1 or 2 prominent nucleoli, and pathological mitoses at high magnification (thick arrow) (Fig. 3F). Morphologically, melanoma in situ of nasal mucosa and its transition to the epithelioid area of the tumor in submucosa were observed (Fig. 3G and H).

### Immunohistochemical results

Immunohistochemical detection of S-P method was performed on sections to further confirm the nature of the neoplasm.

**Fig. 4:**
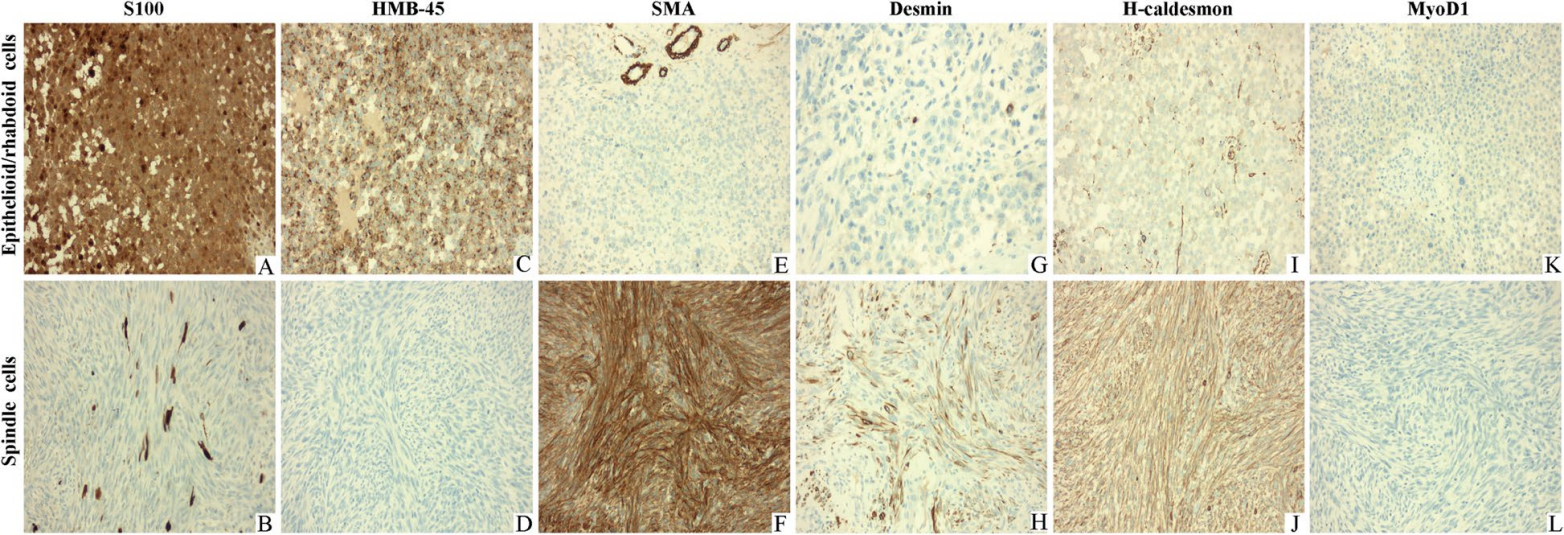
The epithelioid or rhabdoid tumor cells showed strongly and diffusely immunoreactivity for S100 (**A**, × 200) and HMB-45 (**C**, × 200). However, the spindle tumor components were scatteredly positive for S100 (**B**, × 200) and negative for HMB-45 (**D**, × 200). a-SMA was negative in the epithelioid or rhabdoid neoplastic cells (**E**, × 200) and diffusely positive in the spindle neoplastic cells (**F**, × 200). Desmin was focally positive in the epithelioid or rhabdoid neoplastic cells (**G**, × 400), and partially diffuse positivity in the spindle-shaped tumor cells (**H**, × 200). H-caldesmon showed focal reactivity in the epithelioid or rhabdoid neoplastic cells (**I**, × 200) and diffuse reactivity in the spindle neoplastic cells (**J**, × 200). MyoD1 was negative in all neoplastic cells (**K** and **L**, × 200)

The neoplastic cells of the epithelioid or rhabdoid areas were diffusely positive for melanocytic markers of S100 (Fig. 4A), HMB-45 (Fig. 4C), SOX10, Melan-A and PNL2, whereas only scattered cells of the spindle components were positive for S100 (Fig. 4B) and SOX10. In the spindle neoplastic cells areas, a-SMA and H-caldesmon were diffusely positive (Fig. 4F and J). Desmin was negative for most of the neoplastic cells and partially was diffuse positive (Fig. 4H). However, in the epithelioid or rhabdoid areas, the neoplastic cells exhibited negative for a-SMA (Fig. 4E), and focally positive for H-caldesmon (Fig. 4I) and Desmin (Fig. 4G). All tumor cells were positive for INI-1, H3K27me3 and failed to express MyoD1 (Fig. 4K and L), myogenin and all epithelial markers of CK, CK7, EMA (Table 1).

**Table 1:** Immunohistochemical staining results of the tumor cells

Antibody	Manufacture	Dilution	Epithelioid/rhabdoid cells	Clone	Spindle cells
S100	MXB	Predilute	+	4C4.9	Scattered individually+
SOX10	MXB	Predilute	+	EP268	Scattered individually+
HMB-45	MXB	Predilute	+	HMB-45	–
Melan-A	MXB	Predilute	+	A103	–
PNL2	MXB	Predilute	+	PNL2	–
a-SMA	MXB	Predilute	–	MX097	+
Desmin	MXB	Predilute	Focal +	MX046	Partially diffuse+
H-caldesmon	MXB	Predilute	Focal +	H-CALD	+
Calponin	MXB	Predilute	–	MX023	Focal +
MyoD1	MXB	Predilute	–	MX049	–
Myogenin	MXB	Predilute	–	MX078	–
Pan-CK	MXB	Predilute	–	AE1/AE3	–
CK7	MXB	Predilute	–	MX053	–
EMA	MXB	Predilute	–	E29	–
INI-1	MXB	Predilute	+	MRQ-27	+
H3K27me3	ZSGB-BIO	Predilute	+	RM175	+

Abbreviations: *MXB* Fuzhou Maixin Biotechnology, Fujian, China, *ZSGB-BIO* Zhongshanjinqiao, Beijing, China

In light of these radiological, histological and immunohistochemical findings, a diagnosis of primary SNMM with smooth muscle differentiation was established.

## Discussion

MM usually was comfirmed very difficultly because of multiple morphological changes. It occasionally shows particular differentiation, leading to more diagnostic challenges. MM with smooth muscle differentiation is extraordinary unwonted, with no more than 4 previously reportorial cases [11–14]. These reported 4 cases were all female, with the age between 54 yrs. and 73 yrs., and belonged to cutaneous melanoma (Table 2). As summarized in Table 2, this type of tumor was more prone to amelanotic, which might cause confusion in diagnosis. The neoplasm components in all these 4 cases were positive for markers of both melanin and smooth muscle by immunohistochemistry. Herein, we reported a case of SNMM with smooth muscle differentiation. Smooth muscle differentiation was comfirmed with the positive immunostaining for a-SMA, H-caldesmon, calponin and Desmin. The main differential diagnosis of histopathology of this presented case included other biphasic neoplasms, such as biphasic synovial sarcoma, malignant peripheral nerve sheath tumor (MPNST) with epithelioid or rhabdomyosarcomatous differentiation, epithelioid sarcoma, melanotic perivascular epithelioid cell neoplasm (PEComa), as well as sarcomatoid carcinoma.

**Table 2:** Clinicopathological characteristics of reported cases of MM with smooth muscle cell differentiation (Including Current Case)

Reference	Age/Sex	Sites	Cell morphology	Pigment	Melanocyctic markers	Other markers	Differentiation
Banerjee et al. 1996 [11]	54/F	The epigastric area and right axillary lymph node metastasis, the subcutaneous tissue of the left upper back, right inguinal nodes metastasis and metastasis around the iliac vessels	Plump spindle cells, polygonal cells and large numbers of multinucleated tumour giant cells	Absent	S100+, HMB-45-	a-SMA+, Desmin+, HHF-35+	Smooth muscle
Ul-Mulk et al. 2012 [12]	73/F	Right arm, right axillary nodes and multiple metastases to the liver, lung, breast and skin	Composed of two different contiguous morphological, respectively melanosomes and leiomyosarcoma	Present	S100+, Melan A+	a-SMA+, Desmin+	Smooth muscle
Morimoto et al. 2014 [13]	63/F	The left toe and left groin lymph node metastasis	The neoplasm was composed of pleomorphic spindle cells	Absent	S100+, MITF-1+, HMB-45+	a-SMA+, Desmin+	Smooth muscle
Prieto-Torres et al. 2017 [14]	69/F	The left scapular area	The tumor cells with rhabdoid and spindle morphology	Absent	S100+, SOX10+, Melan A-, HMB-45-	a-SMA+, Desmin+	Smooth muscle
Current case	54/M	The right nasal cavity	Epithelioid/rhabdoid and spindle cells	Present	S100+, SOX10+, Melan A+, HMB-45+, PNL2+	a-SMA+, Desmin+, Caldesmon+, Cloponin+	Smooth muscle

Synovial sarcoma is a peculiar, malignant and aggressive high grade soft neoplasm that arises at any age but more common in teenagers and young adults [15]. This neoplasm was classified histopathologically into types of monophasic, biphasic and poorly differentiated [16]. Biphasic synovial sarcoma may resemble the current case because neoplastic cells may exhibit epithelioid and spindle-shaped morphologies [17]. But most biphasic synovial sarcoma shows immunostain for the cytokeratins (particularly CK7 and Pan-CK), and EMA in the epithelioid components [18]. Nonetheless, the presented case showed positive for more specific melanocyte markers of HMB-45, Melan-A, SOX10, PNL2, and negative for three epithelial markers of CK7, Pan-CK and EMA, favoring the diagnosis of MM.

Another notable morphologically similar lesion was MPNST with epithelioid or rhabdomyosarcomatous differentiation. The latter also was known as malignant Triton tumor. Malignant Triton tumor often exhibits immunoreactivity for specific striated muscle markers [19], which displayed reactionless in current case. Furthermore, a history of neurofibromatosis type 1 would be a strong indicator of MPNST over MM [20]. Previous studies had implied that deficiency of H3K27 trimethylation (H3K27me3) happened in up to half of MPNST and it may be a specific diagnostic clue for MPNST [21, 22], but H3K27me3 was positive in this case.

Epithelioid sarcoma was other differential diagnoses in histomorphology. The epithelioid sarcoma tumor cells lost the expression of INI-1 [23], but INI-1 was diffusely immunoreactive in this case.

Melanotic PEComa is also a particularly rare but confusing differential diagnosis that must be taken into account, as it can present as an epithelioid or spindle-shaped morphology and harbour melanin pigmentation similar to the case [24]. This type of tumor regularly expresses melanocytic markers such as HMB45 and Melan A, but much less rarely expresses S100 or SOX10, which are always positive in melanomas with an epithelioid or spindle-shaped morphology. This may therefore be informative in the differential diagnosis.

In addition, SNMM should be distinguished from sarcomatoid carcinoma because of a biphasic pattern. The epithelial markers were negatively in the tumor cells favored for the diagnosis of MM. Moreover, metastatic MM needed to be excluded before making the diagnosis of primary SNMM because of the rarity of primary SNMM compared to cutaneous MM. A whole-body PET/CT and histologically melanoma in situ were helpful for ruling out metastatic disease.

In conclusion, we put forward a highly peculiar, but edifying case of SNMM with areas of smooth muscle differentiation, which might be a possible pathological diagnostic pitfall. Unambiguous immumohistochemical staining for both melanocytic and smooth muscle markers was essential for its correct diagnosis. The clinical significance of this rare variation in morphology is unclear, further cases are urgently required to explore its association with prognosis. Understanding of divergent histological patterns of MM is beneficial to avert the risk for misdiagnosis of these extraordinary and aggressive neoplasms.

## Data Availability

All data generated or analyzed during this presented case are included within this article.

## Abbreviations

SNMM: Sinonasal Mucosal Melanoma
MM: Malignant melanoma
CT: Computed tomography
PET/CT: Positron emission tomography/computed tomography
MPNST: Malignant peripheral nerve sheath tumor
PEComa: Perivascular epithelioid cell neoplasm
